# Exploring Consumers’ Negative Electronic Word-of-Mouth of 5 Military Hospitals in Taiwan Through SERVQUAL and Flower of Services: Web Scraping Analysis

**DOI:** 10.2196/54334

**Published:** 2024-05-29

**Authors:** Ching-Yuan Huang, Po-Chun Lee, Long-Hui Chen

**Affiliations:** 1 Department of Marketing Management SHU-TE University Kaohsiung Taiwan; 2 Department of Internal Medicine Kaohsiung Armed Forces General Hospital Kaohsiung Taiwan; 3 Institute of Medical Science and Technology National Sun Yat-sen University Kaohsiung Taiwan; 4 General Education Center, R.O.C Naval Academy Kaohsiung Taiwan; 5 Department of Business Management National Kaohsiung Normal University Kaohsiung Taiwan

**Keywords:** electronic word-of-mouth, eWOM, service quality, SERVQUAL scale, Flower of Services, health care service quality, military hospitals

## Abstract

**Background:**

In recent years, with the widespread use of the internet, the influence of electronic word-of-mouth (eWOM) has been increasingly recognized, particularly the significance of negative eWOM, which has surpassed positive eWOM in importance. Such reviews play a pivotal role in research related to service industry management, particularly in intangible service sectors such as hospitals, where they have become a reference point for improving service quality.

**Objective:**

This study comprehensively collected negative eWOM from 5 military hospitals in Taiwan that were at or above the level of regional teaching hospitals. It aimed to investigate service quality issues before and after the pandemic. The findings provide important references for formulating strategies to improve service quality.

**Methods:**

In this study, we used web scraping techniques to gather 1259 valid negative eWOM, covering the period from the inception of the first review to December 31, 2022. These reviews were categorized using content analysis based on the modified Parasuraman, Zeithaml, and Berry service quality (PZB SERVQUAL) scale and Flower of Services. Statistical data analysis was conducted to investigate the performance of service quality.

**Results:**

The annual count of negative reviews for each hospital has exhibited a consistent upward trajectory over the years, with a more pronounced increase following the onset of the pandemic. In the analysis, among the 5 dimensions of PZB SERVQUAL framework, the “Assurance” dimension yielded the least favorable results, registering a negative review rate as high as 58.3%. Closely trailing, the “Responsiveness” dimension recorded a negative review rate of 34.2%. When evaluating the service process, the subitem “In Service: Diagnosis/Examination/Medical/Hospitalization” exhibited the least satisfactory performance, with a negative review rate of 46.2%. This was followed by the subitem “In Service: Pre-diagnosis Waiting,” which had a negative review rate of 20.2%. To evaluate the average scores of negative reviews before and during the onset of the COVID-19 pandemic, independent sample *t* tests (2-tailed) were used. The analysis revealed statistically significant differences (*P*<.001). Furthermore, an ANOVA was conducted to investigate whether the length of the negative reviews impacted their ratings, which also showed significant differences (*P*=.01).

**Conclusions:**

Before and during the pandemic, there were significant differences in evaluating hospital services, and a higher word count in negative reviews indicated greater dissatisfaction with the service. Therefore, it is recommended that hospitals establish more comprehensive service quality management mechanisms, carefully respond to negative reviews, and categorize significant service deficiencies as critical events to prevent a decrease in overall service quality. Furthermore, during the service process, customers are particularly concerned about the attitude and responsiveness of health care personnel in the treatment process. Therefore, hospitals should enhance training and management in this area.

## Introduction

### Background

With the internet’s and social media’s rapid development in recent years, electronic word-of-mouth (eWOM) has become consumers’ preferred mechanisms for evaluating the service quality performance of businesses and organizations, as well as for communication. Numerous academic studies have consistently demonstrated the profound impact of eWOM on consumers’ long-term decision-making, surpassing the influence of traditional marketing efforts and media exposure. Consequently, it can be inferred that negative eWOM is more likely to sway consumer decisions than positive eWOM and may even pose a substantial threat to the sustained operations and growth of service-oriented organizations [1-10].

According to the service characteristics elaborated by Zeithaml [11], health care services fall into the category of the most intangible and challenging-to-evaluate services, necessitating a high degree of trust in service providers. Several scholars have highlighted the heightened importance of eWOM for intangible goods compared with conventional tangible products [12-16]. Consequently, eWOM wield a significant influence on consumer decision-making, particularly when it comes to health care institutions. In the increasingly competitive landscape of the health care service industry, patients encounter various forms of service during their medical visits. These encounters shape their perceptions of health care institutions and, subsequently, their inclination to engage in word-of-mouth (WOM) communication. Taiwan’s health care system is tiered into medical centers, regional hospitals, district hospitals, and primary clinics. This study specifically gathered data from Google Maps reviews (out of 5 points) of all 19 medical centers, 88 regional hospitals, and the major 5 regional-level military hospitals across Taiwan. The findings revealed that the average rating was 3.4 for medical centers, 3.27 for regional hospitals, and 3.08 for military hospitals, indicating that military hospitals’ ratings were lower than those of comparable civilian hospitals. However, on the basis of our comprehensive review and search, only Lee et al [17] have explored both positive and negative eWOM within the context of military health care institutions. Furthermore, their study was confined to a single health care institution, providing somewhat restricted insights into the broader comprehension of eWOM within the hospital sector. In addition, Perrault and Hildenbrand [10] conducted experiments to determine how consumer exposure to negative web-based reviews and subsequent access to primary health care providers’ eWOM biographies can influence patient decisions and mitigate the effects of negative reviews.

Therefore, this study uses web scraping techniques to collect eWOM data from military hospitals in Taiwan with regional teaching levels or higher across various regions. Content analysis will be conducted, categorizing and summarizing the data based on the modified SERVQUAL scale by Parasuraman et al [18] and Flower of Services by Hashem [19]. Subsequently, statistical analysis would be carried out to identify the performance of each service quality dimension and negative feedback within the Flower of Services framework. This study comprehensively collected negative eWOM evaluations of 5 military hospitals in Taiwan. The research aimed to explore issues related to service quality (SERVQUAL) and service processes (based on Flower of Services) that emerged before and during the pandemic. On the basis of the research results, recommendations for strategies to improve service quality will be formulated.

### Literature Review

Due to the prevalence of the internet, many people use eWOM to assess and express the service quality of hospitals. eWOM has a significant impact on consumer decision-making regarding health care institutions. Therefore, the literature review will explain the 3 research theories used in this study: Parasuraman, Zeithaml, and Berry (PZB) SERVQUAL scale, Flower of Services, and research related to eWOM.

### Service Quality

**Figure 1:**
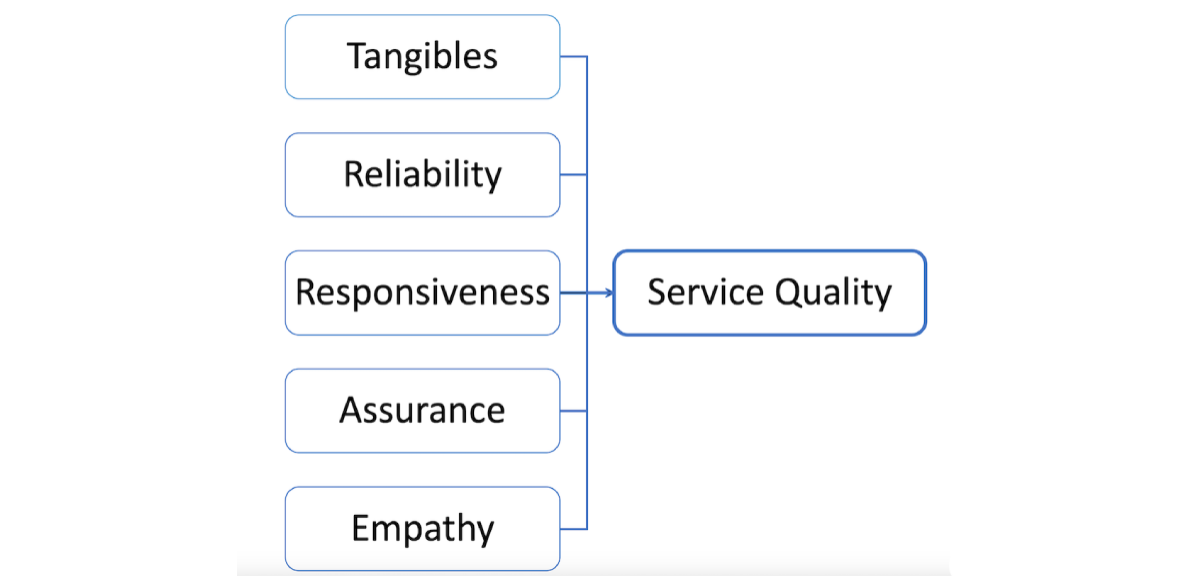
Service quality (SERVQUAL) scale diagram.

Service quality refers to the level of excellence achieved in the service delivery process and interactions between service providers and customers. With the advent of modern technology and improvements in residents’ living standards, many customers now consider service quality as a crucial factor when making consumption decisions, surpassing mere consideration of product pricing. The conceptual model of service quality, originally proposed by Parasuraman et al [20] and known as PZB model, places customers at the center of the service quality equation. In 1988, the PZB model underwent further refinement and simplification, condensing the original 10 dimensions and 97 items into 5 dimensions and 22 items, giving rise to “SERVQUAL” scale. The variables within SERVQUAL scale encompass tangibility, reliability, responsiveness, assurance, and empathy. Figure 1 provides a visual representation of SERVQUAL theoretical model.

Parasuraman et al [18] undertook a redefinition and restructuring of the 5 dimensions of SERVQUAL scale, which are also the measurement dimensions of service quality used in the assessment of negative eWOM of health care institutions within this study [18].

Below are the definitions of each dimension according to Parasuraman et al [18].

1. Tangibles: pertain to physical facilities, equipment, and the appearance of service personnel, among other aspects.
2. Reliability: this signifies the capacity to consistently deliver promised services in a dependable manner.
3. Responsiveness: involves the willingness to assist customers promptly and provide them with timely help.
4. Assurance: focuses on the competence and courtesy exhibited by service personnel, thereby fostering trust and confidence among customers.
5. Empathy: encompasses the provision of caring and personalized attention to customers.

The Parasuraman, Zeithaml, and Berry service quality (PZB SERVQUAL) model holds widespread recognition in the realm of service quality research due to its well-documented reliability and validity, making it a frequently applied tool across diverse industries. As observed by Jonkisz et al [21], results obtained through the SERVQUAL method hold the potential to aid in enhancing and monitoring service quality across various organizations. Notably, the health care sector extensively uses the SERVQUAL method. A study demonstrated its utility in evaluating service quality within intensive care units (ICUs) [22]. Their research revealed that ICUs generally perform satisfactorily across most dimensions, aligning with the perceptions of patients and their families. This highlights the method’s capacity to offer crucial insights into ICU service quality, facilitating enhancements in patient care. Moreover, the assessment of hospital health care service quality plays a pivotal role in advancing health care systems. An illustrative example is the study conducted within a large public university hospital center in Croatia [23]. They applied the SERVQUAL method to analyze the significance of different service quality dimensions and the disparities between patients’ perceptions and expectations concerning health care services across various departments. The results unveiled substantial gaps in service quality dimensions, with “responsiveness” and “tangibles” exhibiting the most pronounced disparities. This underscores the need for management’s focused efforts in addressing these facets to augment service quality. Furthermore, the research conducted by Malathi and Jasim [24] highlighted that patients prioritize various service quality factors, including reliability, tangibility, empathy, responsiveness, and assurance, when selecting health care services through medical applications. However, in the context of applying SERVQUAL theory to eWOM-related research, Pauli et al [25] analyzed a total of 34 studies on WOM in health care from January 2000 to December 2019. Their focus was primarily on applying the theory of cognitive dissonance, the theory of the strength of weak ties, and the theory of perceived risk, with no cases integrating SERVQUAL theory. In our search within the “Health Care Sciences & Services” field, only Lee et al [17] studied positive and negative WOM at a regional teaching hospital in Taiwan. They found that during the COVID-19 pandemic, negative reviews exceeded positive ones, with “Assurance” performing the worst in negative eWOM among the 5 determinants of PZB SERVQUAL, followed by “Responsiveness” and “Reliability” [17]. Since the study was limited to one hospital, its extrapolation to all health care institutions is insufficient. Therefore, our research expands the scope to comprehensively collect eWOM over the years from 5 representative military hospitals above the level of regional teaching hospitals in different regions of Taiwan. By examining a larger sample size and the broader population, we aimed to validate the explanatory power of the SERVQUAL theory applied to negative eWOM.

### Flower of Services

**Figure 2:**
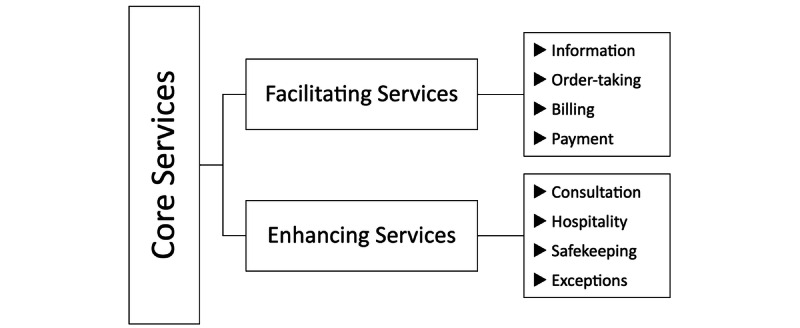
Flower of Services.

The Flower of Services, proposed by Lovelock and Wirtz [26], summarizes the interactions between supplementary services in facilitating or enhancing services within the core product. It comprises 8 petals, each representing an essential process of physical service. The 8 petals are *Information*, *Consultation*, *Order taking*, *Hospitality*, *Safekeeping*, *Exceptions*, *Billing*, and *Payment*. Information, Order taking, Billing, and Payment fall under the category of facilitating services aimed at assisting the use of the core product and emphasizing the correctness of the service. Consultation, Hospitality, Safekeeping, and Exceptions are categorized as enhancing services, which increase the service value and create differentiation. The more the resources invested, the higher the service value can be improved (Figure 2). The research findings of Hashem [19] suggest that the Information, Consultation, Order taking, Hospitality, and Billing dimensions of Flower of Services have a significant impact on customer satisfaction, while Safekeeping and Exceptions do not seem to influence it [19]. In this study, the 11 service process items of Flower of Services have been adapted and expanded according to the general medical service processes of hospitals, and they are categorized into 3 stages: before service (4 items), in-service (5 items), and after service (2 items).

### Negative eWOM

As defined by Harrison-Walker [27], WOM communication refers to informal interpersonal communication, encompassing interactions between noncommercial communicators and potential disseminators. With the rapid advancement of internet technology, consumers increasingly tend to share their opinions on products, brands, and companies through various internet-based platforms, known as eWOM or internet WOM [28,29]. WOM can be categorized into positive and negative forms, with negative WOM often stemming from consumer dissatisfaction, where individuals share their discontent or raise complaints about specific products or services [30].

According to the research conducted by Filieri et al [31], eWOM has become one of the most persuasive sources of information for consumer decision-making. Consumers frequently proactively seek eWOM related to products and services to aid them in making purchase decisions. The study by Purcarea et al [32] focused on the credibility of eWOM information in the context of health care services and found that argument strength had the most significant impact on intention.

Past research on web-based consumer behaviors has indicated that consumers place a higher importance on negative eWOM than positive eWOM, suggesting that negative eWOM has a more significant influence on consumer decisions than positive eWOM [1-10]. Pauli et al [25] analyzed WOM in health care by reviewing 34 studies conducted between January 2000 and December 2019. Their research highlighted the significance of staff during service interactions and revealed that negative reviews have a greater impact than positive ones. Service quality was identified as a primary cause of negative WOM. The study also emphasized the growing popularity and importance of eWOM in patients’ decision-making processes [25]. Nadarajan et al [4] further indicated that the more dissatisfied a consumer is, the greater the number of negative words, images, and emoticons in eWOM. Strongly worded negative eWOM is more likely to impact consumer behavior. Izogo et al [33] suggested that consumers’ prior web-based shopping experiences influence their perception of negative eWOM posted on social media, implying that negative reviews have a more significant impact on new customers, as existing customers have past consumption experiences to reference in their decision-making. Moreover, eWOM during COVID-19 was notably characterized by low trust and high perceived risk [34]. The research conducted by Lee et al [17] showed that during the COVID-19 pandemic, negative reviews surpassed positive reviews.

## Methods

### Data Collection

Big data analytics has become an effective method for modern organizations to identify and solve operational and business performance issues. Specifically, a wealth of data is available on the internet, especially on social media sites and internet-based platforms, which organizations can effectively use. Using web scraping techniques to mine data from relevant internet-based platforms can greatly aid organizational management applications. This study follows the basic web scraping design process steps proposed by Lotfi et al [35].

- Step 1, querying the web using seed URLs: select Google Maps reviews of 5 military hospitals above the level of teaching hospitals in Taiwan as specific point of interest (POI).
- Step 2, crawl through the web using seed URLs: extract the corresponding URLs from the aforementioned specific POIs.
- Step 3, parse the website’s contents and extract the required content from crawled URLs: use Python’s Beautiful Soup library to parse the content of the corresponding websites and extract the comment-related content required for the study.
- Step 4, store the extracted content into a database: save the extracted data in a CSV file.

Finally, by analyzing the CSV file, we can gain valuable insights into consumer sentiment, identify common themes and patterns, and measure overall satisfaction with the POIs. In summary, this study selected Google Maps consumer reviews of 5 military hospitals above the level of teaching hospitals across Taiwan as the research subject. It used web scraping techniques designed with the Python programming language to capture eWOM data from the first eWOM until December 31, 2022. After filtering, we obtained 1259 valid negative eWOM data entries. These review data were then categorized and analyzed using the modified PZB SERVQUAL scale and Flower of Services. Subsequently, a statistical analysis of service quality performance and service processes was conducted. SPSS (version 20.0; IBM Corp) statistical software was used for descriptive statistics, independent sample *t* tests, and ANOVA analysis to compare differences in service quality indicators and service processes before and during the pandemic.

### Ethical Considerations

The data for this study were sourced from publicly available web-based information, and the study was approved by the institutional review board of Antai Medical Cooperation, Antai Tian-Sheng Memorial Hospital (TSMH institutional review board number and protocol number: 23-079-C). The informed consent form was not required, as the obtained data had been deidentified, ensuring no privacy or confidentiality issues. The participants of the study were not test participants, and there were no issues regarding compensation.

### Variable Measures

According to SERVQUAL scale, these items are divided into 5 dimensions (refer to Textbox 1 for details) [17]. On the basis of the comprehensive hospital service process, the original 8 processes of Flower of Services are modified into 9 service processes [26]. These are divided into 4 preservice processes and 5 in-service processes corresponding to the 8 service processes; and 2 postservice processes are added. This results in 3 major stages encompassing 11 processes (refer to Table 1 for details).

**The classification items for the 5 dimensions of the SERVQUAL questionnaire (the items are indicated in English as the first letter of each of the 5 dimensions).**

**Dimension and items**

- Tangibles
  T1: Insufficiency in hospital public facilities (including transportation, parking, and circulation design)
  T2: Inadequacy of accessibility facilities in the hospital
  T3 Lack of cleanliness, hygiene, and esthetic quality in the hospital environment
  T4: Inadequacy of medical facilities in the hospital and a lack of quiet comfort
  T5: Poor identification of attire among health care personnel
  T6: Other tangible issues (such as website registration service information, gifts, auxiliary facilities, apps, etc)
- Reliability
  R1: Health care personnel are unable to treat medical conditions effectively
  R2: Physicians’ inability to provide detailed information on the medical condition and treatment approach
  R3: Health care personnel are unable to provide medical services promptly
  R4: Other reliability issues (medical falsification: filling out data without conducting examinations)
- Responsiveness
  RE1: Health care personnel cannot promptly respond to service requests (service waiting time)
  RE2: Health care personnel cannot communicate the service process and waiting times
  RE3: Health care personnel do not proactively and willingly assist patients
  RE4: Other responsiveness issues (eg, no response to telephone inquiries, unreachable, or transfers to disconnected lines)
- Assurance
  A1: Health care personnel do not possess professional skills and knowledge
  A2: Health care personnel lack professional service communication attitudes and etiquette
  A3: The hospital’s equipment lacks safety
  A4: Other assurance issues (eg, vaccine shortages leading to cancelations, security collaboration, and poor taxi service attitudes in scheduling)
- Empathy
  E1: Health care personnel are unable to meet individualized service needs
  E2: The hospital does not provide a diverse selection of appointment times
  E3: The hospital does not prioritize the personal privacy of patients
  E4: Other empathy issues (identity discrimination)

**Table 1:** Detailed classification of the 3 major stages of Flower of Services.

Stage	Items	Flower of Services
Before service	B1 website or app search services B2 phone, social media, or app consultation services B3 appointment scheduling services (on the web, phone, or in-person) B4 convenience of transportation and parking facilities or environmental esthetics	Information Consultation Order taking
In service	S1 preappointment waiting S2 examination, checkup, medical, or hospitalization service process S3 billing, including waiting time S4 in-service: medication dispensation, including waiting time and explanations S5 in-service: other additional services	Hospitality Safekeeping Exceptions Billing Payment
After service	A1 posttreatment question consultation and care services A2 follow-up appointment notification and scheduling services	N/A^a^

^a^N/A: not applicable.

## Results

### Analysis of Customer Negative Reviews Over the Years and Trends

**Figure 3:**
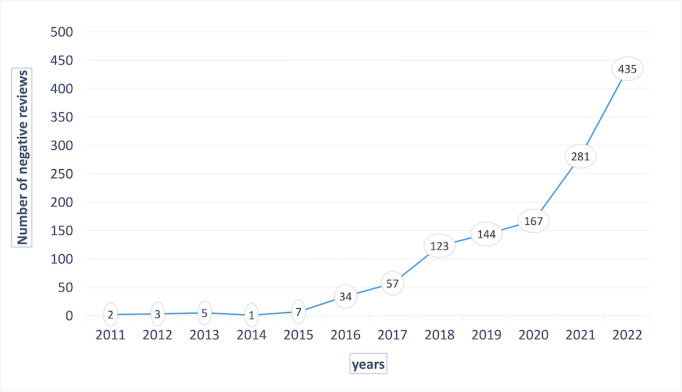
Trend chart of negative reviews over the years.

On the basis of the analysis in Table 2 and Figure 3, we can observe that the total number of eWOM for the 5 hospitals has shown a year-on-year upward trend. Particularly after the outbreak of COVID-19 in 2020, there is a noticeable increase in the slope between 2021 and 2022. This trend suggests that the impact of the COVID-19 pandemic has significantly influenced the current health care policies and internal hospital management.

**Table 2:** Overview of customer negative review counts over the years^a^.

Hospital	Years											
	2011, n (%)	2012, n (%)	2013, n (%)	2014, n (%)	2015, n (%)	2016, n (%)	2017, n (%)	2018, n (%)	2019, n (%)	2020, n (%)	2021, n (%)	2022, n (%)
H1^b^ (n=463)	1 (0.2)	2 (0.4)	2 (0.4)	0 (0)	2 (0.4)	14 (3)	24 (5.2)	56 (12.1)	48 (10.4)	57 (12.3)	98 (21.2)	159 (34.3)
H2^c^ (n=281)	N/A^d^	N/A	N/A	N/A	N/A	3 (1.1)	16 (5.7)	21 (7.5)	32 (11.4)	38 (13.5)	63 (22.4)	108 (38.4)
H3^e^ (n=240)	N/A	N/A	N/A	N/A	4 (1.7)	12 (5)	8 (3.3)	22 (9.2)	32 (13.3)	21 (8.8)	48 (20)	93 (38.8)
H4^f^ (n=215)	1 (0.5)	1 (0.5)	3 (1.4)	1 (0.5)	1 (0.5)	5 (2.3)	9 (4.2)	23 (10.7)	28 (13)	40 (18.6)	49 (22.8)	54 (25.1)
H5^g^ (n=60)	N/A	N/A	N/A	N/A	N/A	N/A	N/A	1 (1.7)	4 (6.7)	11 (18.3)	23 (38.3)	21 (35)

^a^Total (n=1259); year 2011, n=2; year 2012, n=3; year 2013, n=5; year 2014, n=1; year 2015, n=7; year 2016, n=34; year 2017, n=57; year 2018, n=123; year 2019, n=144; year 2020, n=167; year 2021, n=281; year 2022, n=435.

^b^Tri-Service General Hospital.

^c^Taoyuan Armed Forces General Hospital.

^d^N/A: not applicable.

^e^Taichung Armed Forces General Hospital.

^f^Kaohsiung Armed Forces General Hospital.

^g^Hualien Armed Forces General Hospital.

### Statistical Analysis of Negative Ratings

Negative reviews received the lowest rating of 1 star, accounting for 81.97% (1032/1259) of the total reviews. Therefore, it can be inferred that when individuals are extremely dissatisfied with hospital services, they are more likely to proactively leave negative ratings and written comments on the web (Table 3).

**Table 3:** Frequency and percentage of negative ratings^a^.

Hospital	Points^b^, n (%)		
	1 point	2 points	3 points
H1^c^ (n=463)	358 (77.3)	66 (14.3)	39 (8.4)
H2^d^ (n=281)	247 (87.9)	19 (6.8)	15 (5.3)
H3^e^ (n=240)	199 (82.9)	23 (9.6)	18 (7.5)
H4^f^ (n=215)	174 (80.9)	24 (11.2)	17 (7.9)
H5^g^ (n=60)	54 (89.3)	4 (7.1)	2 (3.6)

^a^Total (n=1259); 1 point, n=1032; 2 points, n=136; 3 points, n=91.

^b^Star ratings on Google Maps range from 1 to 5 stars, with a corresponding rating scale of 1 to 5 points. Both 1 and 2 stars are categorized as negative reviews. For reviews with a rating of 3 stars, the sentiment is determined based on the content of the review. If there is no textual review accompanying a 3-star rating, it is categorized as a positive review.

^c^Tri-Service General Hospital.

^d^Taoyuan Armed Forces General Hospital.

^e^Taichung Armed Forces General Hospital.

^f^Kaohsiung Armed Forces General Hospital.

^g^Hualien Armed Forces General Hospital.

### Analysis of Severity Levels of Negative Reviews

As shown in Table 4, of the 1259 comments, samples (n=239, 19%) with no written comments for negative reviews were excluded. The remaining negative reviews totaled 1020 (81.97%) samples. Among these were 404 (39.6%) samples in categories A and B that had comments exceeding 51 words, representing the total negative reviews and indicating the percentage of individuals who were willing and proactive in leaving comments and reviews when they perceived shortcomings in hospital services.

**Table 4:** Analysis of severity levels of negative reviews^a^.

Hospital	Rating			
	A (reviews >101 words), n (%)	B (reviews approximately 51-101 words), n (%)	C (reviews <50 words), n (%)	D (only ratings, no reviews), n (%)
H1^b^, n=463	59 (12.7)	79 (17.1)	228 (49.2)	97 (21)
H2^c^, n=281	44 (15.7)	46 (16.4)	153 (54.4)	38 (13.5)
H3^d^, n=240	26 (10.8)	40 (16.7)	113 (47.1)	61 (25.4)
H4^e^, n=215	45 (20.9)	52 (24.2)	89 (41.4)	29 (13.5)
H5^f^, n=60	4 (6.7)	9 (15)	33 (55)	14 (23.3)

^a^The total negative reviews excluding (D) samples with only ratings and no comments for the 5 hospitals amount to 1020 negative reviews. Total (n=1259); rating A, n=178; rating B, n=226; rating C, n=616; rating D, n=239.

^b^Tri-Service General Hospital.

^c^Taoyuan Armed Forces General Hospital.

^d^Taichung Armed Forces General Hospital.

^e^Kaohsiung Armed Forces General Hospital.

^f^Hualien Armed Forces General Hospital.

### Negative Analysis of the 5 SERVQUAL Dimension

#### Analysis of Negative Rating Frequency and Percentage for the 5 Service Quality Dimensions

Table 5 provides a valuable insight into the distribution of the frequency of negative comments (n=1020). Notably, the dimension labeled “Assurance” emerges as the most prominent, with a staggering 58.3% (n=595) of the negative comments. Close on its heels, the “Responsiveness” dimension registers registered at 34.2% (n=349), while “Empathy” ranks as the least frequently encountered dimension, accounting for only 10.7% (n=109) of the reviews. For a more granular perspective, when evaluating individual hospitals, it becomes evident that H4 boasts the highest frequency of negative comments within the “Assurance” dimension, reaching a noteworthy 64% (119/186) of the reviews. Conversely, H3 takes the lead in the “Responsiveness” dimension, with a substantial frequency of 36.9% (66/179) of the reviews.

**Table 5:** Negative ratings frequency and percentage for the 5 service quality dimensions^a^.

Hospital	Dimensions					
	Tangibles, n (%)	Reliability, n (%)	Responsiveness, n (%)	Assurance, n (%)	Empathy, n (%)	Total, n (%)
H1^b^ (n=366)	*89* (24.3)	68 (18.6)	133 (36.3)	206 (56.3)	39 (10.7)	*535* (146.2)
H2^c^ (n=243)	26 (10.7)	21 (8.6)	81 (33.3)	136 (56)	13 (5.3)	277 (114)
H3^d^ (n=179)	18 (10.1)	24 (13.4)	66 (36.9)	108 (60.3)	15 (8.4)	231 (129.1)
H4^e^ (n=186)	43 (23.1)	51 (27.4)	55 (29.6)	119 (64)	33 (17.7)	301 (161.8)
H5^f^ (n=46)	5 (10.9)	11 (23.9)	14 (30.4)	26 (56.5)	9 (19.6)	65 (141.3)

^a^Each negative comment may cover >1 dimension. n represents the total number of individuals who left negative comments about the hospitals. Total (n=1020); tangibles, n=181; reliability, n=175; responsiveness, n=349; assurance, n=595; empathy, n=109.

^b^Tri-Service General Hospital.

^c^Taoyuan Armed Forces General Hospital.

^d^Taichung Armed Forces General Hospital.

^e^Kaohsiung Armed Forces General Hospital.

^f^Hualien Armed Forces General Hospital.

#### Analysis of the Frequency and Percentage of Negative Subindicators for SERVQUAL’s 5 Dimensions

Table 6 provides significant insights into the distribution of negative reviews within the “Tangibles” dimension. Notably, the highest percentage of negative reviews, 44.2% (80/181), is attributed to “T1 Hospital public facilities are inadequate.” Following closely, “T6 Other tangible facilities” accounts for 26.5% (48/181) of the reviews, while the lowest percentage, a mere 1.1% (2/181) of the reviews, corresponds to “T2 Hospital lacks accessible facilities.” Delving into individual hospitals, it is evident that H1 has the highest percentage of negative reviews regarding “T1 Hospital public facilities are inadequate,” standing at 54% (48/89) of the reviews. This underscores the critical role played by hospital public facilities and other tangible services in shaping patient evaluations. Specifically, the evaluation of hospital public facilities is an integral component of the overall patient experience, intricately linked to patient comfort and convenience within the hospital environment. These findings serve as a poignant reminder to hospital administrators that, in their pursuit of improved service quality, they must not only prioritize the professionalism of health care staff but also elevate their focus on enhancing public facilities and other tangible services.

**Table 6:** Frequency and percentage of negative subindicators for SERVQUAL’s 5 dimensions.

Dimensions and items^a^			Hospital										
			H1^b^, n (%)		H2^c^, n (%)		H3^d^, n (%)		H4^e^, n (%)		H5^f^, n (%)		Total, n (%)
**Tangibles (H1, n=89, H2, n=26, H3, n=18, H4, n=43, H5, n=5; Σn=181)**													
	T1	48 (53.9)		12 (46.2)		7 (38.9)		13 (30.2)		0 (0)		80 (44.2)	
	T2	1 (1.1)		0 (0)		0 (0)		1 (2.3)		0 (0)		2 (1.1)	
	T3	15 (16.9)		7 (26.9)		2 (11.1)		8 (18.6)		0 (0)		32 (17.7)	
	T4	7 (7.9)		3 (11.5)		3 (16.7)		18 (41.9)		1 (20)		32 (17.7)	
	T5	2 (2.2)		0 (0)		1 (5.6)		2 (4.7)		0 (0)		(2.8)	
	T6	25 (28.1)		5 (19.2)		6 (33.3)		8 (18.6)		4 (80)		48 (26.5)	
**Reliability (H1, n=68; H2, n=21; H3, n=24; H4, n=51; H5, n=11; Σn=175)**													
	R1	41 (60)		7 (33.3)		7 (29.2)		31 (60.8)		4 (30)		90 (51.4)	
	R2	37 (54.4)		8 (38.1)		7 (29.2)		10 (19.6)		0 (0)		62 (35.4)	
	R3	39 (57.4)		11 (52.4)		8 (33.3)		17 (33.3)		10 (90)		85 (48.6)	
	R4	3 (4.4)		1 (4.8)		2 (8.3)		3 (5.9)		0 (0)		9 (5.1)	
**Responsiveness (H1, n=133; H2, n=81; H3, n=66; H4, n=55; H5, n=14; Σn=349)**													
	RE1	79 (59.4)		43 (53.1)		39 (59.1)		42 (76.4)		9 (61.5)		212 (60.7)	
	RE2	78 (58.6)		42 (51.9)		33 (50)		26 (47.3)		6 (46.2)		185 (53)	
	RE3	72 (54.1)		6 (7.4)		6 (9.1)		7 (12.7)		3 (23.1)		94 (26.9)	
	RE4	7 (5.3)		10 (12.3)		4 (6.1)		4 (7.3)		0 (0)		25 (7.2)	
**Assurance (H1, n=206; H2, n=136; H3, n=108; H4, n=119; H5, n=26; Σn=595)**													
	A1	71 (34.5)		29 (21.3)		28 (25.9)		43 (36.1)		4 (12.5)		175 (29.4)	
	A2	182 (88.3)		112 (82.4)		86 (79.6)		79 (66.4)		22 (87.5)		481 (80.8)	
	A3	1 (0.5)		0 (0)		3 (2.8)		1 (0.8)		0 (0)		5 (0.8)	
	A4	7 (3.4)		7 (5.1)		2 (1.9)		8 (6.7)		1 (4.2)		25 (4.2)	
**Empathy (H1, n=39, H2, n=13; H3, n=15; H4, n=33; H5, n=9; Σn=109)**													
	E1	10 (25.6)		5 (38.5)		2 (13.3)		16 (48.5)		6 (66.7)		39 (35.8)	
	E2	2 (5.1)		1 (7.7)		0 (0)		1 (3)		1 (11.1)		5 (4.6)	
	E3	5 (12.8)		0 (0)		2 (13.3)		2 (6.1)		2 (22.2)		11 (10.1)	
	E4	26 (66.7)		7 (53.8)		11 (73.3)		12 (36.4)		0 (0)		56 (51.4)	

^a^The code “T” stands for Tangibles subitems, “R” for Reliability subitems, “RE” for Responsiveness subitems, “A” for Assurance subitems, and “E” for Empathy subitems.

^b^Tri-Service General Hospital.

^c^Taoyuan Armed Forces General Hospital.

^d^Taichung Armed Forces General Hospital.

^e^Kaohsiung Armed Forces General Hospital.

^f^Hualien Armed Forces General Hospital.

Within the “Reliability” dimension, an analysis of the total negative reviews revealed significant patterns. Specifically, 51.4% (90/175) of the negative reviews revolved around “R1: Healthcare staff did not effectively treat the condition,” making it the most prominent concern. Closely trailing, “R3: Healthcare staff did not provide medical services promptly” represented 48.6% (85/175) of the negative reviews, while “R4: Other reliability issues” constituted a minority share of 5.1% (9/175) of the negative reviews. When examining individual hospitals, it becomes evident that H4 exhibited the highest percentage of negative reviews related to “R1: Health care staff did not effectively treat the condition,” standing at 61% (31/51) of the reviews. This underscores the pivotal nature of a hospital’s ability to address patients’ treatment issues effectively. Patients hold robust expectations regarding the professional competence of health care staff and the prompt delivery of medical services, highlighting the imperative role of hospital administrators in prioritizing these dimensions during service quality enhancements. This prioritization ensures that patients receive medical care not only promptly but also with the requisite efficacy, aligning with their elevated expectations.

Within the “Responsiveness” dimension, the highest total of negative reviews was attributed to “RE1: Healthcare staff did not provide service requests promptly,” accounting for 60.7% (212/349) of the reviews. Subsequently, “RE2: Healthcare staff did not inform about the service process and waiting time” followed closely with 53% (185/349) of the reviews, while “RE4: Other responsiveness issues” constituted the lowest percentage, at 7.2% (25/349) of the reviews. Among individual hospitals, H1 exhibited the highest percentage of negative reviews related to “RE1: Healthcare staff did not provide service requests promptly,” standing at 59.4% (79/133) of the negative reviews. This underscores that “service waiting time” is the primary factor influencing the service quality dimension of “Responsiveness.” Patients have elevated expectations regarding whether health care staff can provide services promptly and inform them about waiting times during the medical service waiting process. Consequently, when enhancing service quality, hospital administrators should give special attention to the management of service waiting times to ensure that patients can experience effective responsiveness and care during their wait.

Within the “Assurance” dimension, the highest total of negative reviews was attributed to “A2: Healthcare staff lacking a professional service attitude and etiquette,” accounting for 80.8% (481/595 of the negative reviews. Subsequently, “A1: Healthcare staff lacking professional skills and knowledge” followed at 29.4% (175/595) of the negative reviews, while “A3: Hospital facilities lacking safety” represented the lowest percentage, at 0.8% (5/595) of the negative reviews. Among individual hospitals, H1 exhibited the highest percentage of negative reviews related to “A2: Healthcare staff lacking a professional service attitude and etiquette,” standing at 88.3% (182/206) of the negative reviews. These findings underscore the critical importance of the professional attitude and skills of hospital health care staff when enhancing service quality within the “Assurance” dimension. Patients have exceptionally high expectations regarding the professionalism and courtesy of health care staff, which directly influences their overall assessment of the hospital’s service quality. Consequently, hospital administrators should prioritize professional training and attitude development for health care staff to enhance performance within the “Assurance” dimension.

Within the “Empathy” dimension, the highest total of negative reviews pertains to “E4: Other empathy issues,” accounting for 51.4% (56/109) of the negative reviews. Subsequently, “E1: Healthcare staff unable to meet individual service needs” followed at 35.8% (39/109) of the negative reviews, while “E2: Hospital’s clinic hours do not offer diverse choices” represents the lowest percentage, at 4.6% (5/109) of the negative reviews. Among individual hospitals, H1 exhibited the highest percentage of negative reviews related to “E4: Other empathy issues,” at 67% (26/39) of the negative reviews. When endeavoring to enhance service quality within the “Empathy” dimension, hospitals should emphasize 2 primary aspects. First, they should ensure that health care staff can adapt to the diverse needs of various patients and provide individualized services. Second, it is imperative to prevent any discriminatory behaviors during interactions with patients, ensuring empathetic and compassionate communication. These concerted efforts can effectively address concerns related to “Empathy,” elevate overall service quality, and enhance patient satisfaction.

#### Analysis of Negative Reviews in the Service Aspects

On the basis of the data presented in Table 7, which outlines negative evaluations during different stages of service processes, it is evident that the most pivotal juncture in medical services, “S2 During Service: Examination/Treatment/Hospitalization Process,” commands the highest proportion at 46.2% (471/1020). Following closely is “S1 During Service: Pre-Examination Waiting,” comprising 20.2% (206/100) of the total. Upon examining individual hospitals, it becomes apparent that H4 exhibits the highest proportion for “S2 During Service: Examination/Treatment/Hospitalization Process,” at 53.8% (100/186). Conversely, “S1 During Service: Pre-Examination Waiting” holds the highest proportion at H5, accounting for 39% (18/46).

**Table 7:** Negative feedback status at various stages of the service process (Flower of Services)^a^.

Hospital	Stages											
	B1, n (%)	B2, n (%)	B3, n (%)	B4, n (%)	S1, n (%)	S2, n (%)	S3, n (%)	S4, n (%)	S5, n (%)	A1, n (%)	A2, n (%)	Total, n (%)
H1^b^ (n=366)	1 (0.3)	13 (3.6)	17 (4.6)	15 (4.1)	51 (13.9)	158 (43.2)	10 (2.7)	3 (0.8)	6 (1.6)	5 (1.4)	5 (1.4)	284 (77.6)
H2^c^ (n=243)	1 (0.4)	12 (4.9)	15 (6.2)	24 (9.9)	59 (24.3)	121 (49.8)	1 (0.4)	5 (2.1)	3 (1.2)	3 (1.2)	2 (0.8)	246 (101.2)
H3^d^ (n=179)	1 (0.6)	2 (1.1)	5 (2.8)	2 (1.1)	38 (21.2)	77 (43)	4 (2.2)	2 (1.1)	1 (0.6)	3 (1.7)	1 (0.6)	136 (76)
H4^e^ (n=186)	8 (4.3)	7 (3.8)	8 (4.3)	13 (7)	40 (21.5)	100 (53.8)	3 (1.6)	2 (1.1)	7 (3.8)	1 (0.5)	3 (1.6)	192 (103.2)
H5^f^ (n=46)	0 (0)	1 (2.2)	5 (10.9)	1 (2.2)	18 (39.1)	15 (32.6)	0 (0)	0 (0)	2 (4.3)	1 (2.2)	1 (2.2)	44 (95.7)

^a^Codes B, S, and A represent before service, during service, and after service, respectively. Total (n=1020); stage B1, n=11; stage B2, n=35; stage B3, n=50; stage B4, n=55; stage S1, n=206; stage S2, n=471; stage S3, n=18; stage S4, n=12; stage S5, n=19; stage A1, n=13; stage A2, n=12.

^b^Tri-Service General Hospital.

^c^Taoyuan Armed Forces General Hospital.

^d^Taichung Armed Forces General Hospital.

^e^Kaohsiung Armed Forces General Hospital.

^f^Hualien Armed Forces General Hospital.

These findings underscore the substantial influence of service encounters, especially during critical moments of truth, and the impact of service waiting times on patient satisfaction. In addition, according to Flower of Services, 3 out of the 4 enhancing factors (Figure 2), namely, Hospitality, Safekeeping, and Exceptions, predominantly fall under the “in service” phase (Table 1). This corroborates the findings of this study with the original theoretical implications, further emphasizing the importance of S1 and S2 in the medical service process.

### Comparative Mean Analysis

#### Independent Sample t test Before and During the COVID-19 Pandemic

An independent sample *t* test was carried out to assess the variance in negative ratings before and after the onset of the COVID-19 pandemic. As indicated in Table 8, a noteworthy distinction in negative ratings before and after the pandemic is evident (*P*<.001). The mean negative rating following the pandemic (mean 1.21, SD 0.539) is notably lower than the period preceding the pandemic (mean 1.34). This observation underscores the substantial and adverse influence of the pandemic on the quality of hospital services.

**Table 8:** Differences in negative review severity with respect to negative ratings^a^.

Prepandemic or during pandemic		Number of reviews	Values, mean (SD)	*F* test (*df*)		*P* value	
**Negative review score**					40.52 (1257)		<.001
	Prepandemic	376	1.34 (0.651)				
	During pandemic	883	1.21 (0.539)				

^a^The distinction between prepandemic and postpandemic scores is determined based on the announcement by the Ministry of Health and Welfare of Taiwan on January 15, 2020, declaring “Severe Acute Respiratory Syndrome Coronavirus 2 (SARS-CoV-2) infection,” commonly known as COVID-19, as a Class V Notifiable Infectious Disease.

#### ANOVA on the Severity of Negative Reviews and Negative Ratings

An ANOVA was conducted to explore the distinctions in negative review severity concerning negative ratings. As presented in Table 9, significant differences in negative review severity relative to negative ratings were observed (*P*<.05). Further post hoc Scheffe tests unveiled discernible intergroup variations. In essence, the degree of negative review text severity considerably contributed to the variation in negative ratings. Notably, the average ratings for negative reviews exhibited a diminishing pattern in tandem with increasing word count, specifically A (mean 1.20)<B (mean 1.21)<C (mean 1.31). This indicates that as the negative review content becomes more comprehensive and lengthier, the corresponding negative ratings tend to decrease.

**Table 9:** Differences in negative review severity on negative ratings (n=1259)^a^.

			Values, n (%)		Values, mean (SD)		*F* test (*df*)			*P* value^b^	
**Word count of reviews**								3.596 (1258)			.01
	A (reviews >101 words)	178 (14.1)		1.20 (0.525)							
	B (reviews approximately 51-101 words)	226 (18)		1.21 (0.538)							
	C (reviews <50 words)	616 (48.9)		1.31 (0.641)							
	D (only ratings, no reviews)	236 (18.7)		1.19 (0.454)							

^a^Dependent variables: negative review scores (*P*<.05 significance).

^b^Post hoc Scheffe tests revealed the presence of intergroup differences**.**

## Discussion

### Principal Findings

This study used web mining techniques to collect eWOM data from Google Maps, spanning from the initial review to December 31, 2022. Out of the collected data, 1259 negative eWOM were initially obtained. After the exclusion of 239 samples lacking accompanying text, the final data set comprised 1020 negative reviews. The statistical analysis unveiled a notable upsurge in negative eWOM during 2021 and 2022, following the emergence of the COVID-19 pandemic in 2020. This surge in negative reviews can be ascribed to various uncontrollable factors stemming from the pandemic, leading to issues and service deficiencies. For instance, concerns regarding vaccine distribution prioritization, vaccine supply shortages, hospital crowd management, bed allocation, and staffing challenges may have contributed to patient and family dissatisfaction. These concerns manifested as negative reviews, potentially impacting service quality. This observation aligns with the findings of Lee et al [17] and Nilashi et al [34].

Furthermore, the analysis revealed that most negative scores were rated at 1 point, encompassing 81.97% (1032/1259) of the total negative reviews. Notably, reviews categorized as A and B, which contained ≥51 words, accounted for 39.6% (404/1020) of all negative reviews. This suggests that when patients encounter significant service deficiencies, particularly severe ones, their dissatisfaction is notably pronounced. In such situations, individuals may feel compelled to articulate their experiences, emotions, and encountered issues in more extensive and detailed reviews.

In situations of heightened severity, patients may feel inclined to provide elaborate narratives, aiming to enhance hospitals’ or others’ understanding of their predicaments. This is often done in the hope of garnering more attention and triggering remedial actions. Consequently, lengthier reviews can be seen as reflections of patients’ emotional expressions when confronted with substantial service deficiencies and their earnest desire for service improvements. This study used semantic content analysis to categorize comments based on the modified PZB SERVQUAL scale, followed by rigorous statistical analysis. The research outcomes unveiled that the most significant proportion of negative comments pertained to “Assurance,” constituting 58.33% (595/1020) of the reviews, followed closely by “Responsiveness” at 34.2% (349/1020) of the reviews. These findings align seamlessly with the observations made by Lee et al [17]. These data underscore the pivotal role of the “Tangibles” aspect, particularly the professionalism and demeanor of health care personnel, in shaping patients’ perceptions of service quality within hospital settings. Furthermore, secondary factors influencing these evaluations encompass the “Responsiveness” of the hospital, including service waiting times and the transparency of the service process. This analysis accentuates the profound impact of health care personnel’s conduct on patients’ experiences and satisfaction within a medical milieu, with service timeliness and clarity emerging as additional influential factors shaping overall evaluations.

In terms of specific subitems, the top 3 concerns were as follows: “A2: Lack of professionalism and courtesy from healthcare personnel” at 80.8% (481/595), followed by “RE1: Failure of healthcare personnel to provide timely service requests” at 60.7% (212/349). Tied for the third position were “R1: Failure of healthcare personnel to effectively treat the condition” and “E4: Other empathy issues,” both at 51.4% (56/109). This suggests that the public places the utmost importance on 3 dimensions of service quality: the “Professionalism and courtesy of healthcare personnel and their ability to provide timely service requests,” “Failure of healthcare personnel to effectively treat the condition,” and “Other empathy issues (such as identity discrimination).” These findings are in alignment with those presented by Lee et al [17].

This underscores the fact that in their health care experiences, individuals consider the professionalism of health care personnel, the promptness of service delivery, and the efficacy of treatments as pivotal factors in their assessment of a hospital’s overall quality. Consequently, in the pursuit of augmenting overall service quality, hospitals should direct their efforts toward enhancing these aspects. First and foremost, the cultivation and refinement of health care personnel’s professionalism and courtesy hold paramount importance. This encompasses the enhancement of their professional knowledge and skills, communication abilities, interpersonal relationships, and responsiveness to patient needs. Second, ensuring that health care personnel promptly and efficiently address patient service requests is vital for bolstering patient confidence and contentment. Finally, hospitals should continually optimize medical procedures and treatment methodologies to guarantee that patients receive effective care, thereby elevating the overall quality of health care services. In addition, considering the role of rank awareness in military hospital services warrants further exploration.

When analyzing negative feedback across different stages of the service journey, it becomes evident that the most crucial juncture in medical services, labeled as “S2 In-Service: Consultation/Examination/Medical Treatment/Hospitalization Process,” accounted for the highest proportion at 46.18% (471/1020). Following closely, “S1 In-Service: PreConsultation Waiting” constituted 20.2% (206/1020). These findings underscore 2 pivotal aspects: first, the moments of direct service interaction, often referred to as the “Moment of Truth” in health care services, significantly influence patient satisfaction. Consequently, in the pursuit of elevating overall service quality, hospitals should dedicate particular attention to enhancing performance during this phase. Second, the entirety of the waiting period within the health care process carries substantial importance for patients. Therefore, hospitals should endeavor to reduce waiting times and implement more efficient waiting management strategies (eg, using technological solutions such as the Right Time appointment scheduling app). In summary, this study demonstrates that improving health care service quality entails not only ensuring the proficient execution of medical procedures but also focusing on effective time management throughout the process to guarantee that patients receive more satisfactory and well-coordinated care.

An independent sample *t* test was conducted to assess differences in negative ratings before and after the COVID-19 pandemic. The analysis revealed a significant disparity in negative ratings before and after the pandemic (*P*<.001), with postpandemic negative ratings (mean 1.21, SD 0.539) being notably lower than their prepandemic counterparts (mean 1.34, SD 0.651). This suggests that the COVID-19 pandemic substantially influenced customer evaluations of service quality. The outbreak of the pandemic appears to have reshaped perceptions and expectations regarding service quality, highlighting the challenges it posed to hospital operations and service delivery. These findings align with the conclusions reached by Lee et al [17] and Nilashi et al [34].

In the final phase of this study, an ANOVA was conducted to explore variations in negative rating scores concerning the severity of negative comments, as detailed in Table 9. The outcomes unveiled a significant distinction in negative rating scores associated with the severity of negative comments (*P*<.05). However, post hoc Scheffe tests did not disclose noteworthy distinctions among the groups. In essence, the intensity of language used in negative comments appears to play a pivotal role in determining the extent of negative rating scores. It is imperative to acknowledge that as the word count within negative comments escalated, the average negative rating scores exhibited a descending pattern, with category A (mean 1.20, SD 0.525)<category B (mean 1.21, SD 0.538)<category C (mean 1.31, SD 0.641). This indicates that patients tend to furnish more comprehensive narratives in their negative comments, particularly when they are more dissatisfied with the institution’s services. This observation aligns with the findings presented by Nadarajan et al [4]. Although the intensity of negative comments did not have a substantial impact on negative rating scores, patients’ willingness to invest more effort in expressing their dissatisfaction or the ability of detailed content to encapsulate specific service grievances could be inferred. This outcome underscores the significance of comprehending and addressing patients’ detailed comments as a means to enhance health care service quality.

### Limitations and Future Directions

This study relied on eWOM from Google Maps. While Google Maps is a widely used platform for such reviews, it may not capture the entire spectrum of patients’ experiences, as some individuals may prefer other review platforms or choose not to leave eWOM at all. In the meantime, this study focused on 5 regional military hospitals in Taiwan, which may limit the generalizability of the findings to other health care settings or regions. Health care systems and patient expectations can vary significantly between countries and regions. The analysis of eWOM, even when categorized systematically, is still subject to interpretation and subjective judgments. Different analysts may categorize reviews differently, potentially leading to variations in results.

While this study categorized reviews based on word count, it did not delve deeply into the qualitative aspects of the reviews. Future research could explore the specific content of longer reviews to gain more insights into patients’ concerns and suggestions. Future research could include more in-depth qualitative analysis of the content within the eWOM. This would provide a richer understanding of patients’ experiences and allow for the identification of specific areas for improvement. Furthermore, comparative studies could be conducted to assess service quality across different types of health care institutions, such as public versus private hospitals or military versus civilian health care facilities. This could shed light on variations in patient experiences.

Hospitals could implement interventions based on the findings of this study and assess their impact on service quality and patient satisfaction. This would provide practical insights into improving health care services. In addition, hospitals should consider integrating eWOM feedback into their quality improvement processes. Establishing mechanisms for systematically addressing patient concerns and suggestions can lead to continuous service quality enhancement.

Finally, given the cultural variations in patient expectations and communication styles, cross-cultural studies could compare eWOM and service quality perceptions in different countries or regions. Studies should be conducted to develop predictive models that can anticipate shifts in service quality perceptions based on factors such as changes in health care policies, staff training programs, or external events such as pandemics. Given the sensitivity of health care data, future research should also consider issues related to data security and patient privacy, especially when analyzing eWOM.

### Conclusions

In an era where eWOM wield immense influence over consumer decisions, this study has delved into eWOM to analyze service quality perceptions in the context of military health care institutions in Taiwan. The findings shed light on several crucial aspects of service quality, providing valuable insights for health care administrators and policy makers.

However, it is essential to consider the limitations of this study, including its reliance on Google Maps reviews, which may not capture all patient experiences, and its focus on military health care institutions in Taiwan, limiting generalizability.

In addition, this study not only highlights the power of eWOM in shaping perceptions but also provides actionable insights for health care institutions. It emphasizes the need to focus on professionalism, responsiveness, and critical service encounters, especially in the postpandemic era when health care expectations and priorities may have evolved. By addressing these dimensions and continuously seeking to improve service quality, hospitals can better meet patient needs, enhance patient satisfaction, and ultimately provide higher-quality health care services.

## Abbreviations

eWOM: electronic word-of-mouth
ICU: intensive care unit
POI: point of interest
PZB SERVQUAL: Parasuraman, Zeithaml, and Berry service quality
WOM: word-of-mouth

## Data Availability

The data are available upon request.

## References

[1] Luo MM, Chien CC (2021). Factors affecting negative EWOM: a literature review and merged model. Proceedings of the 54th Hawaii International Conference on System Sciences.

[2] Melander T, Dyrelöv F (2021). The impact of negative ewom on brand loyalty: a qualitative study in the context of social media. Department of Business Administration, Umeå School of Business and Economics.

[3] Israeli AA, Lee SA, Bolden EC (2019). The impact of escalating service failures and internet addiction behavior on young and older customers' negative eWOM. J Hosp Tourism Manage.

[4] Nadarajan G, Bojei J, Khalid H (2017). The study on negative eWOM and its relationship to consumer’s intention to switch mobile service provider. Procedia Comput Sci.

[5] Kim SJ, Wang RJ, Maslowska E, Malthouse EC (2016). “Understanding a fury in your words”: the effects of posting and viewing electronic negative word-of-mouth on purchase behaviors. Comput Human Behav.

[6] Cheung CM, Thadani DR (2012). The impact of electronic word-of-mouth communication: a literature analysis and integrative model. Decis Support Syst.

[7] Christodoulides G, Michaelidou N, Argyriou E (2012). Cross-national differences in e-WOM influence. Eur J Mark.

[8] Lee M, Rodgers S, Kim M (2009). Effects of valence and extremity of eWOM on attitude toward the brand and website. J Curr Issues Res Advert.

[9] Lee M, Youn S (2015). Electronic word of mouth (eWOM): how eWOM platforms influence consumer product judgement. Int J Advert.

[10] Perrault EK, Hildenbrand GM (2020). The buffering effect of health care provider video biographies when viewed in combination with negative reviews: "you can't fake nice". J Med Internet Res.

[11] Zeithaml VA, Donnelly JH, George WR (1981). How consumer evaluation processes differ between goods and services. Marketing of Services.

[12] Zhou S, Guo B (2017). The order effect on online review helpfulness: a social influence perspective. Decis Support Syst.

[13] Liu Z, Park S (2015). What makes a useful online review? Implication for travel product websites. Tour Manag.

[14] Sotiriadis MD, van Zyl C (2013). Electronic word-of-mouth and online reviews in tourism services: the use of Twitter by tourists. Electron Commer Res.

[15] Karmarkar UR, Tormala ZL (2010). Believe me, I have no idea what I’m talking about: the effects of source certainty on consumer involvement and persuasion. J Consum Res.

[16] Ye Q, Law R, Gu B (2009). The impact of online user reviews on hotel room sales. Int J Hosp Manag.

[17] Lee PC, Liang LL, Huang MM, Huang CY (2022). A comparative study of positive and negative electronic word-of-mouth on the SERVQUAL scale during the COVID-19 epidemic - taking a regional teaching hospital in Taiwan as an example. BMC Health Serv Res.

[18] Parasuraman A, Zeithaml VA, Berry LL (1988). Servqual: a multiple-item scale for measuring consumer perceptions of service quality. J Retailing.

[19] Hashem TN (2018). The flower of service concept and its influence on the customer satisfaction: case study of Jordanian private hospitals sector. Int J Bus Manag.

[20] Parasuraman A, Zeithaml VA, Berry LL (1985). A conceptual model of service quality and its implications for future research. J Mark.

[21] Jonkisz A, Karniej P, Krasowska D (2022). The servqual method as an assessment tool of the quality of medical services in selected Asian countries. Int J Environ Res Public Health.

[22] Lu SJ, Kao HO, Chang BL, Gong SI, Liu SM, Ku SC, Jerng JS (2020). Identification of quality gaps in healthcare services using the SERVQUAL instrument and importance-performance analysis in medical intensive care: a prospective study at a medical center in Taiwan. BMC Health Serv Res.

[23] Ozretić Došen D, Škare V, Čerfalvi V, Benceković Ž, Komarac T (2020). Assessment of the quality of public hospital healthcare services by using SERVQUAL. Acta Clin Croat.

[24] Malathi A, Jasim KM (2022). Validating the relationship between service quality, patient sensitivity and experience towards medical applications using SERVQUAL. Int J Med Inform.

[25] Pauli G, Martin S, Greiling D (2022). The current state of research of word-of-mouth in the health care sector. Int Rev Public Nonprofit Mark.

[26] Lovelock CH, Wirtz J (2011). Services Marketing: People Technology Strategy.

[27] Harrison-Walker LJ (2016). The measurement of word-of-mouth communication and an investigation of service quality and customer commitment as potential antecedents. J Serv Res.

[28] Yang L, Cheng Q, Tong S, Yang H, Morgan SL, Wang Y (2015). Empirical study of eWOM’s influence on consumers’ purchase decisions. The Strategies of China's Firms: Resolving Dilemmas.

[29] Gu D, Yang X, Li X, Jain HK, Liang C (2018). Understanding the role of mobile internet-based health services on patient satisfaction and word-of-mouth. Int J Environ Res Public Health.

[30] Singh J (1990). Voice, exit, and negative word-of-mouth behaviors: an investigation across three service categories. J Acad Mark Sci.

[31] Filieri R, Galati F, Raguseo E (2021). The impact of service attributes and category on eWOM helpfulness: an investigation of extremely negative and positive ratings using latent semantic analytics and regression analysis. Comput Human Behav.

[32] Purcarea VL, Gheorghe IR, Petrescu CM (2013). Credibility elements of eWOM messages in the context of health care services. A Romanian perspective. J Med Life.

[33] Izogo EE, Jayawardhena C, Karjaluoto H (2022). Negative eWOM and perceived credibility: a potent mix in consumer relationships. Int J Retail Distrib Manag.

[34] Nilashi M, Ali Abumalloh R, Alrizq M, Alghamdi A, Samad S, Almulihi A, Althobaiti MM, Yousoof Ismail M, Mohd S (2022). What is the impact of eWOM in social network sites on travel decision-making during the COVID-19 outbreak? A two-stage methodology. Telemat Inform.

[35] Lotfi C, Srinivasan S, Ertz M, Latrou I (2021). Web scraping techniques and applications: a literature review. Proceedings of the 2nd Congress on Intelligent Systems.

